# Adaptive Control Method for Gait Detection and Classification Devices with Inertial Measurement Unit

**DOI:** 10.3390/s23146638

**Published:** 2023-07-24

**Authors:** Hyeonjong Kim, Ji-Won Kim, Junghyuk Ko

**Affiliations:** 1 Division of Mechanical Engineering, (National) Korea Maritime and Ocean University, Busan 49112, Republic of Korea; 2 Division of Biomedical Engineering, Konkuk University, Chungju 27478, Republic of Korea; 3 BK21 Plus Research Institute of Biomedical Engineering, Konkuk University, Seoul 05029, Republic of Korea

**Keywords:** adaptive control, control algorithm, cueing training, feedback training, gait assessment gait classification, gait detection, gait evaluation, Parkinson’s disease, rehabilitation

## Abstract

Cueing and feedback training can be effective in maintaining or improving gait in individuals with Parkinson’s disease. We previously designed a rehabilitation assist device that can detect and classify a user’s gait at only the swing phase of the gait cycle, for the ease of data processing. In this study, we analyzed the impact of various factors in a gait detection algorithm on the gait detection and classification rate (GDCR). We collected acceleration and angular velocity data from 25 participants (1 male and 24 females with an average age of 62 ± 6 years) using our device and analyzed the data using statistical methods. Based on these results, we developed an adaptive GDCR control algorithm using several equations and functions. We tested the algorithm under various virtual exercise scenarios using two control methods, based on acceleration and angular velocity, and found that the acceleration threshold was more effective in controlling the GDCR (average Spearman correlation −0.9996, *p* < 0.001) than the gyroscopic threshold. Our adaptive control algorithm was more effective in maintaining the target GDCR than the other algorithms (*p* < 0.001) with an average error of 0.10, while other tested methods showed average errors of 0.16 and 0.28. This algorithm has good scalability and can be adapted for future gait detection and classification applications.

## 1. Introduction

### 1.1. Research Background and Related Literatures

Parkinson’s disease (PD) is a neurodegenerative disorder that is caused by a deficiency in dopamine. The main symptoms of PD can be classified as either non-motor or motor. Non-motor symptoms include things like depression and insomnia, while motor symptoms include gait disorders and freezing of gait. Music therapy [1], cueing training [2], and feedback training [3] are all methods that have been used to rehabilitate gait disorders in individuals with PD. These training methods can be effective, particularly when used in conjunction with medication or as part of a standalone rehabilitation program [4,5,6,7].

One form of rehabilitation that has been shown to be particularly effective for PD-related gait disorders is augmented feedback training. Many researchers have explored the use of augmented feedback training to improve gait and alleviate gait disorders [8,9,10,11,12,13,14,15,16,17,18,19,20,21,22,23,24]. However, in order to effectively use these training methods, it is necessary to have a gait analysis system that can detect and evaluate a user’s gait. Gait detection is important for determining whether a person is walking or not, while gait classification is necessary for evaluating the quality of the detected gait. Augmented feedback training methods rely on this information to provide appropriate feedback, such as auditory, visual, or tactile signals.

Traditionally, the gold standard for quantitative gait analysis has been optoelectronic stereophotogrammetry. However, this method requires a large laboratory and a significant budget to support its operation and maintenance [25]. An alternative to optoelectronic stereophotogrammetry is the use of inertial measurement units (IMUs). IMUs are portable and easy to use, making them a popular choice for gait analysis in both daily life and rehabilitation settings [26,27]. The use of IMUs for gait analysis is now widespread.

The IMU gait analysis system was previously characterized by multi-point IMU (MIMU) systems [28], which were also validated through various research studies [29,30,31,32,33,34]. Although MIMU systems have demonstrated their superiority, issues with sensor adjustment, calibration, and consistency have limited their adoption in practice [35]. In recent years, single-point IMU (SIMU) devices have gained competitive advantages as medical devices, particularly in the assessment of the severity of Parkinson’s disease, fall risk, and step count [36,37,38,39,40,41].

According to a review of wearable gait detection devices [42], 23 journal papers have used SIMUs on the shank and 25 journal papers have used SIMUs on the foot in an attempt to make SIMUs a popular wearable system for gait analysis. These papers have primarily focused on identifying a user’s gait pattern using artificial intelligence (AI) and machine learning (ML) methods, such as artificial neural networks, extreme learning machines, convolutional neural networks, and long short-term memory [43,44,45,46,47], or rule-based algorithms to detect gait phases such as heel strike, toe-off, and swing phase [48,49,50,51,52].

However, the authors of this paper believed that a simpler gait analysis method was needed to use SIMUs in daily life. AI and ML require a large amount of data to set personal preferences before use, and rule-based gait phase detection algorithms are too comprehensive for our purposes. Therefore, we developed a device that detects and evaluates gait based on acceleration and angular velocity around the ankle joint at the swing phase of gait cycle [53]. This device uses a gait detection and classification (GDC) method to detect and classify a user’s gait based on various factors and the GDC frequency of the device user, known as the gait detection and classification rate (GDCR). However, we found that GDCR can only be roughly controlled and it is necessary to conduct further research to develop an adaptive GDCR control method.

The development of GDCR control methods is also an invaluable part of improving the previous GDC device and method. During feedback training, the results are divided into two categories: success (positive feedback) and failure (negative feedback). Usually, the frequency of provided positive and negative feedback is closely related to the user’s motivation systems in common [54,55,56,57]. Therefore, the adaptive GDCR control method is crucial for preparing for expected and unexpected events that can affect walking speed, such as medication treatment, accidental injury events, or the gradual degradation of motor control ability during rehabilitation, to maintain, enhance, or prevent the user’s motivation.

### 1.2. Summary of the Previous Research

**Figure 1:**
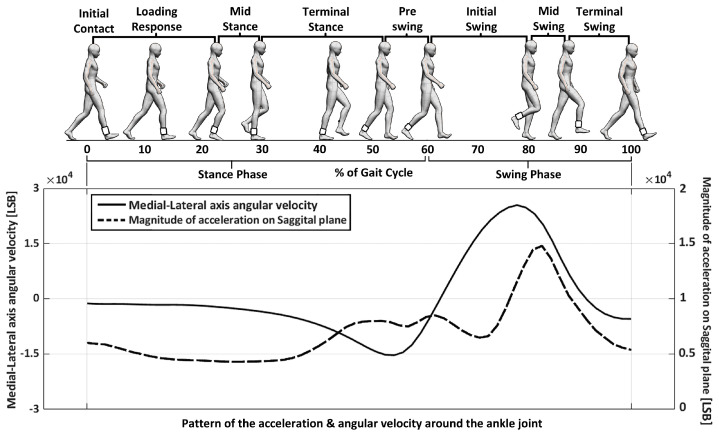
Definition of elements used in previous research to detect and classify a user’s gait.

This research was based on previous work [53] and involved the development of a device for detecting and classifying gait based on angular velocity around the ankle joint during the swing phase of a gait cycle as shown in Figure 1. The device used modified (out of anatomical axis, i.e., directly measured by the device) the medial-lateral axis angular velocity and the magnitude of acceleration on the Sagittal plane around the ankle joint. In what remains of this article, we will loosely use the terms “angular velocity around the ankle” and “ankle acceleration” to refer to the values measured by the IMU. As shown in Figure 1, the factors changed significantly during the swing phase of the gait cycle, so we focused on this phase for gait detection and classification.

The GDC method used a gyroscopic threshold (GT) to detect the user’s gait. If the *z* axis angular velocity of the user was recorded above the GT, the method would detect the user’s gait and set a “gait detection range.” The device also evaluated the user’s gait based on acceleration around the ankle joint using an Acceleration Threshold (AT). If the average of the magnitude of acceleration during the gait detection range was recorded above the AT, the user’s gait would be classified as a “good gait.” The difference between the AT and GT is that the AT is based on the “average value” of data during the gait detection range, while the GT is based on the “value” of the data.

**Figure 2:**
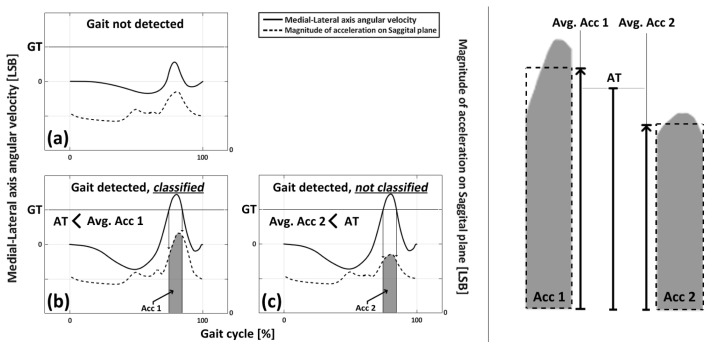
Figure illustrating the role of the AT and GT. (**a**) The case when a gait was not detected (**b**) The case when a gait was detected and classified as a good gait (**c**) The case when a gait was detected but not classified as a good gait.

In the previous research, the AT and GT were used to control the difficulty or ease of gait detection and classification. The GDC method and related devices were used to detect and classify the strength of a user’s gait using the AT and GT as factors. Figure 1 and Figure 2 provide more information on these definitions and how the GDC method works. The GDCR was found to be affected by factors such as walking speed and the presence of gait disorders. The GDCR was also a useful measure for observing and assessing gait conditions.

**Figure 3:**
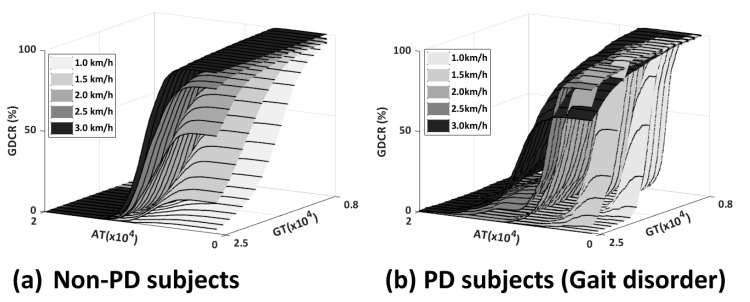
Example results of previous research: (**a**) Results from non-PD subjects; and (**b**) Results from PD subjects.

Twenty-five subjects participated in the assessments of the previous experiment and we collected the acceleration and angular velocity around the ankle joint during one gait cycle. Then, GDC algorithm was applied to the collected data and we observed relations between AT, GT, walking speed, and gait symptom to GDCR. The GDCR was inversely proportional to the AT, GT, and gait disorders, but directly proportional to the walking speed, as shown in Figure 3.

## 2. Materials and Methods

### 2.1. Experiments for Data Collection

Experimental data were collected to develop a GDCR control algorithm and analyze gait. A device that measures acceleration and angular velocity around the ankle joint was used to collect the data. The device was attached to a support band using a metal clip, and subjects were able to wear it themselves, although the experimenter adjusted the devices for better consistency of data. Before the experiment, the devices were calibrated by having subjects stand up straight for 30 s to measure acceleration in their default position. The default position coordinate data were used to calibrate the acceleration. The collected data were processed after the experiment and used to calculate the GDCR at different ATs and GTs. The system provided feedback at every GDC fault.

The subjects included 24 females and 1 male, with an average age of 62 years. None of the subjects had diseases that caused gait disorders. Each subject was asked to walk for one minute at 90 steps per minute cadence (45 times with each leg), with the experimenter providing auditory metronome sounds to indicate the exact timing for walking. Subjects walked three times per each case at treadmill speeds of 1.0, 1.5, 2.0, 2.5, and 3.0 km/h. Subjects took a one minute rest and then walked again, following the sequence walk–rest–walk–rest–walk. If there were more data than 90 steps, only the first 90 steps were used. The experimenter controlled the treadmill speed in ascending order (1 km/h–3 km/h).

**Figure 4:**
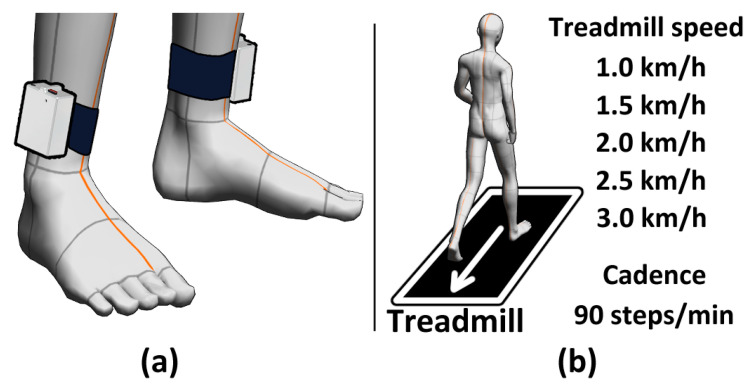
Equipment image and experimental setup: (**a**) equipment example; and (**b**) experimental setup.

The devices used were an MPU-6050 IMU sensor with an ATmega328P chip as the main control unit, and data were collected at a frequency of 30 Hz. 30 Hz was determined to prevent data shifting (phenomenon that some data was recorded at another data line) and data losing (phenomenon that losing IMU data) from low data processing speed. The accelerometers had a sensitivity of 16,384 sensor values per gravity acceleration (9.81 m per second squared (16,384 LSB/(9.81 m/s^2^)), and the gyroscope had a sensitivity of 131 sensor values per degree per second [131 LSB/(deg/s)]. Data generated by the subjects was transmitted via a 2.4 GHz Xbee wireless communication system. Figure 4 shows an image of the equipment and experimental setup.

### 2.2. Impact of the AT and GT on the GDCR

IBM’s SPSS software (SPSS 25) was used to perform statistical analysis to examine the impact of the walking speed, the left and right legs, and the AT and GT on the GDCR. Various statistical methods were used to determine the correlation or difference in the average between each element. Non-parametric tests, the independent sample Mann–Whitney U and Kruskal–Wallis tests, were used to assess the effect of walking speed and the left and right legs on the GDCR. Spearman’s correlation analysis was used to examine the correlation between the AT, GT, and GDCR. The relation between GT and GDCR came by comparing GT and GDCR according to GT at each walking speed. For example, the GT (15,000~25,000) and GDCR (0~1) according to the GT at each walking speed (1.0 km/h~3.0 km/h) was compared. The relation between AT and GDCR came by comparing AT and GDCR according to AT at each walking speed. For example, AT (1000~15,000) and GDCR (0~1) according to the AT at each walking speed (1.0 km/h~3.0 km/h) was compared. Pearson’s correlation analysis was not suitable for this analysis because the experimental dataset did not meet normality. Table 1 provides more details on the statistical analysis.

Twenty-five subjects participated in this research; however, some data on left leg and walking speed 1.0 km/h (*n* = 3) and 2.0 km/h (*n* = 1) were missed. We could compare the impact of walking speed after deleting missing data. However, we could not compute the statistical analysis for the relation between GDCR-GT and GDCR-AT since, for consistency, they can only be compared when we have data for every walking speed.

Number of subjects and samples are different, the number of samples were calculated from the number of subjects, factors, and flaws of data, and we used the number of samples as statistical analysis data. For example, the number of cases of GDCR-Leg relation was 25 on the left leg and 25 on the right leg, but data from 4 participants had flaws on left leg and walking speed 1.0 km/h (*n* = 3) and 2.0 km/h (*n* = 1). Therefore, the number of samples was 121 (cases * walking speeds—flaws, 25 × 5 − 4 = 121) on left and 125 (25 × 5 − 0 = 125) on the right leg. The dependent variable was GDCR, and the independent variable was the leg.

Also, the number of samples of the relation GDCR—walking speed was calculated as follows; the number of subjects × legs (2, left and right) − flaws = the number of samples. The number of samples at 1.0 km/h of walking speed was 47 (25 × 2 − 3 = 47), at 2.0 km/h of walking speed was 49 (25 × 2 − 1 = 49), 50 for other walking speed (25 × 2 − 0 = 50). The dependent variable was GDCR, and independent variable was walking speed.

The number of samples of the relation GDCR-AT was calculated as follows; the number of subjects without flaws of data × the number of AT points of the AT-GDCR curve (1901 points, from 1000 to 20,000, every 10 AT) ((25 − 4) × 1901 = 39,921). The dependent variable was GDCR, and the independent variable was AT. In the case of the relation GT-GDCR, the number of samples is calculated by the number of subjects without flaws of data − the number of GT points of the GT-GDCR curve (21 points, from 15,000 to 25,000, every 500 GT) ((25 − 4) × 21 = 441). Dependent variable was GDCR, independent variable was GT.

### 2.3. Algorithm

The device designed in previous research [53] and improved in this study provided GDC results for the users through various feedback types: visual, auditory, and vibration (somatosensory). The frequency of feedback given when a gait was classified as good is important for gait rehabilitation. The designed rehabilitation device provides feedback whenever a gait fails (when acceleration around the ankle joint cannot be higher than AT). Therefore, the GDCR and the frequency of the gait failure have an inverse relationship. The frequency may vary depending on the rehabilitation method or goal, but if it is too high (>30%) due to high difficulty, users may feel tired and overwhelmed by the device. On the other hand, users may feel that the rehabilitation is insufficient if the frequency is too low (<5%). Therefore, an important feature of the algorithm is its ability to maintain the GDCR, regardless of previous exercise history. Thus, an algorithm was developed that can set the GDCR to be as close as possible to the target GDCR, based on the average GDCR data from 25 subjects. The GDCR target of this algorithm was 0.9 (90% of classified gaits among detected gaits).

**Figure 5:**
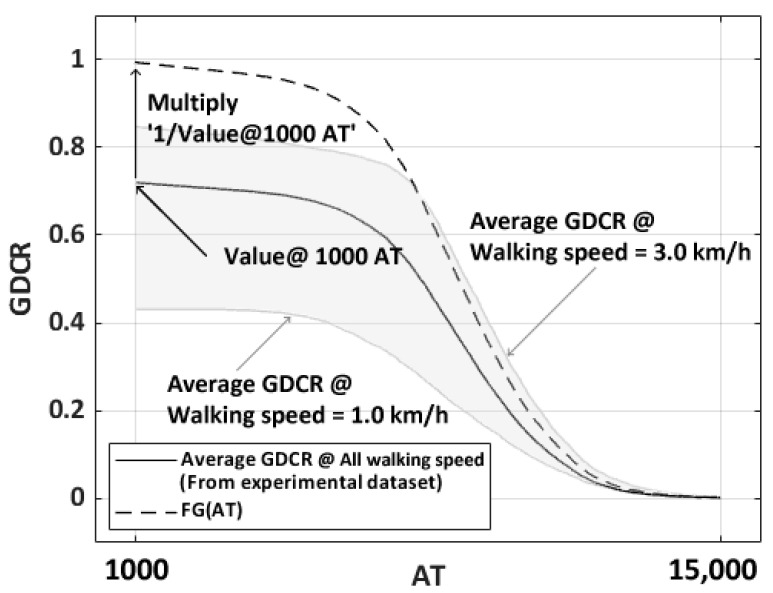
Shape of average GDCR and FG. Solid line curve is the average GDCR from experimental dataset. Dashed line curve which has GDCR range from 0 to 1 is normalized fitted curve from average GDCR curve. Gray-colored area means the average GDCR between slowest (line below of the average GDCR, 1.0 km/h) and fastest (line above of the average GDCR, 3.0 km/h) walking speed.

**Figure 6:**
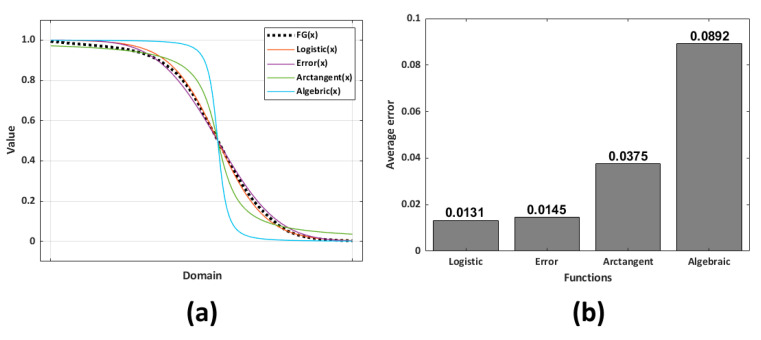
(**a**) FG from the experimental results when GT was 15,000 and various sigmoid functions; and (**b**) average error of sigmoid functions from FG.

The relationship between the AT and the average GDCR of the experimental subjects can be represented as a sigmoid-shaped fitted curve. The function FG was calculated by multiplying the average GDCR from the experimental dataset by 1.38, as shown in Figure 5. The reason of multiplying 1.38 is that to match the range of two different functions: FG and other sigmoid shape functions. The value of 1.38 can be changed into others based on the value of the average GDCR at AT = 1000 (this can be seen in Figure 5). Therefore, the maximum value of FG was 1. In Equation (1), the maximum value of AT was 15,000, and this value was selected based on Figure 5, where it is seen that for high values of AT the GDCR approaches zero. Average GDCR at over 15,000 AT does not significantly affect GDCR. Also, AT at the equation started from 1000 for the same reason. Various sigmoid-shaped functions, such as logistic, error, algebraic, and arctangent functions, were then used to evaluate the similarity to FG. The average error and absolute value of the average differences of these functions with FG were used to determine the similarity. Equations (2)–(5) show the functions used. The average error was calculated using Equation (6). ‘The number of errors’ of Equation (6) was 1400, since the unit of AT was 10 (from 1000 to 15,000, every 10). Figure 6 shows the curves of the various functions and the average errors.

(1) $$FG\left(AT\right)=Normalized\;fitted\;curve\;(1000\leq AT\leq 15,000,0\leq FG\leq 1)$$

(2) $$f_{1}\left(x_{1}\right)=Logistic\left(x_{1}\right)=\frac{1}{1+e^{-x_{1}}}\;(-5.6<x_{1}<7)$$

(3) $$f_{2}\left(x_{2}\right)=Error\left(x_{2}\right)=0.5+\frac{1}{\pi }\int_{0}^{x_{2}}e^{-t^{2}}dt\;(-2.5<x_{2}<2)$$

(4) $$f_{3}\left(x_{3}\right)=Algebraic\left(x_{3}\right)=0.5-\frac{x_{3}}{2\sqrt{1+{x_{3}}^{2}}}\;(-16.5<x_{3}<13)$$

(5) $$f_{4}\left(x_{4}\right)=Arctangent\left(x_{4}\right)=0.5-\frac{1}{\pi }\arctan\left(\frac{\pi x_{4}}{2}\right)\;(-7<x_{4}<5.6)$$

(6) $$Average\;error_{n}=\frac{\sum \left(FG(x)-f_{n}\left(x_{n}\right)\right)}{Number\;of\;errors}\;(1000\leq x\leq 15,000)$$

The logistic function with the least average error of 0.0131 was the function most similar to the FG among the various sigmoid-shaped functions. Therefore, the logistic function’s domain range was converted into the AT domain from 1000 to 15,000 using a trendline, as shown in Equation (7). Subsequently, the function G had converted the x_1_ domain, x_AT_ based on logistic functions such as Equation (8).

(7) $$x_{AT}(AT)=-0.009\cdot \frac{AT-1000}{10}+7.009\;(1000\leq AT\leq 15,000,-5.6\leq x_{AT}\leq 7)$$

(8) $$Logistic\left(AT\right)=G(AT)=\frac{1}{1+e^{0.009\cdot \frac{AT-1000}{10}-7.009}}\;(1000\leq AT\leq 15,000)$$

To determine the features of an individual user, we developed an algorithm that personalizes the GDCR control. The algorithm is based on the average GDCR data from the 25 subjects and is designed to maintain the GDCR regardless of the user’s previous exercise history. The initial personalized coefficient, C_p1_, was calculated as 1.11 times the GDCR at a walking speed of 2 km/h and an AT of 6000. This value was obtained by dividing the average GDCR of experimental data at AT = 1000 by the average GDCR of experimental data at AT = 6000.

For values of n greater than two, C_pn_ can be calculated using Equation (10). The personalized GDCR function, G_p_, can be determined by AT to predict the expected GDCR of each user, as shown in Equation (11). Equation (12) describes the normalized GDCR, G_nor_, which is a function derived from FG. Equation (13) describes the relationship between the AT function and the G function, and Equation (14) is the AT function that calculates the appropriate AT value to achieve the target GDCR, G_target_.

(9) $$C_{p_{1}}=\frac{average\;GDCR\;at\;‘AT=1000’}{average\;GDCR\;at\;‘AT=6000’}\;\mathrm{actual}\;\mathrm{GDCR}\mathrm{at}\;‘\mathrm{AT}=6000’\;\mathrm{and}\;‘2\;\mathrm{km}/h’=1.11\;\mathrm{GDCR}$$

C_p1_ is the initial personalized coefficient. The average GDCR was from all of experimental subjects, and the actual GDCR would be from new experimental data that will be collected from experiments.

(10) $$C_{p_{n}}=\left(average\;GDCR\;at\;AT=1000\right)\cdot \frac{actual\;GDCR}{Expected\;GDCR}=0.720\cdot \frac{actual\;GDCR}{G_{nor}\left(AT\right)}(n\geq 2)$$

C_p2_ is the personalized coefficient to control the algorithm. ‘Expected GDCR’ means expected GDCR with the designed logistic function at current AT and GT. Therefore, G_nor_ can be utilized for the purpose.

(11) $$G_{p}\left(AT\right)=\frac{C_{pn}}{1+e^{0.009\cdot \frac{AT-1000}{10}-7.009}}\left(1000\leq AT\leq 15,000\right)$$

(12) $$G_{nor}(AT)=\frac{average\;GDCR\;at\;AT=1000}{1+e^{0.009\cdot \frac{AT-1000}{10}-7.009}}(1000\leq AT\leq 15,000)$$

The form of G_p_ and G_nor_ was that of the selected sigmoid shape, the logistic function. The x domain of the logistic function changed into ‘0.009 × (AT − 1000)/10 − 7.009’ to utilize the range of AT as a x domain of G_p_ and G_nor_ equations. Also, each equation was multiplied by C_pn_ or ‘average GDCR at AT = 1000’ to change the range of value of the equation.

(13) $$AT(G_{t})=G_{t}^{-1}\left(AT\right)\left(1000\leq AT\leq 15,000,0\leq G_{t}\leq 1\right)$$

(14) $$AT\left(G_{t}\right)=1111.11\cdot \log\left(\left(\frac{C_{p}}{G_{t}}\right)-1\right)+8787.77(0\leq G_{t}\leq 1)$$

AT(G_t_) is the inverse function of G_t_(AT) because G_t_(AT) is defined as the target GDCR according to the current value of AT and its inverted function, AT(G_t_) can mean ‘required AT to reach to G_t_’. G_t_ can be determined from G_p_. This will be described when we introduce the algorithm process.

**Figure 7:**
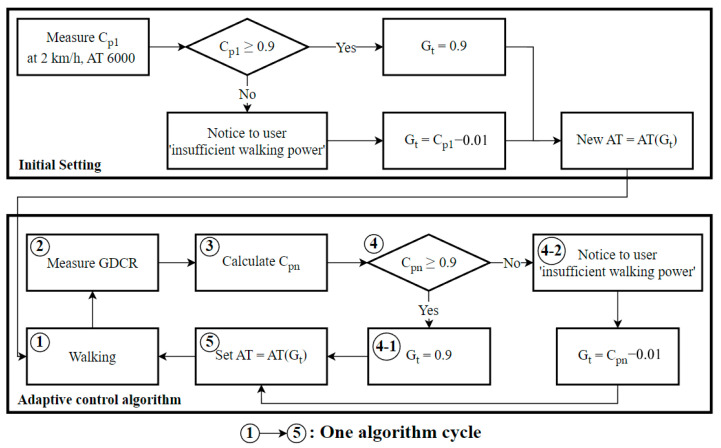
Adaptive control algorithm flowchart.

To design the GDCR adaptive control algorithm using Equation (9) from Equation (14), we followed these steps: (1) Measure C_p1_ at a walking speed of 2 km/h and AT = 6000; (2) Set G_target_ to 0.9 if C_p1_ is greater than 0.9, or set G_target_ to 0.01 less than C_p1_ if C_p1_ is lower than 0.9; (3) Set a new AT at AT(G_target_) (initial setting end); (4) Exercise with the new AT and collect the new GDCR; (5) Calculate the new C_pn_; (6) Set G_target_ to 0.9 if C_pn_ is greater than 0.9, or set G_target_ to 0.01 less than C_pn_ if C_pn_ is lower than 0.9; and (7) Set a new AT with AT(G_target_). Repeat these steps starting at step 4. Figure 7 shows the design process.

This algorithm can lead to confusion surrounding the following question: ‘Will the algorithm classify the first steps as bad and then change the AT value to classify the other steps as ‘good’ since it is an adaptive algorithm?’ However, this adaptive control algorithm is almost a daily-based algorithm. Rather than changing with every step, the idea is that over the workout of a day, for example, about 2 h of walking per day, the results are summarized to obtain an average GDCR and C_pn_ (as shown in Equation (10)) for the day, which is then fed into the algorithm to change for the next day’s or next time’s workout. In this sense, the goal of the algorithm is to keep the frequency of feedback at a similar level while increasing the difficulty so that it can be used for feedback training.

**Figure 8:**
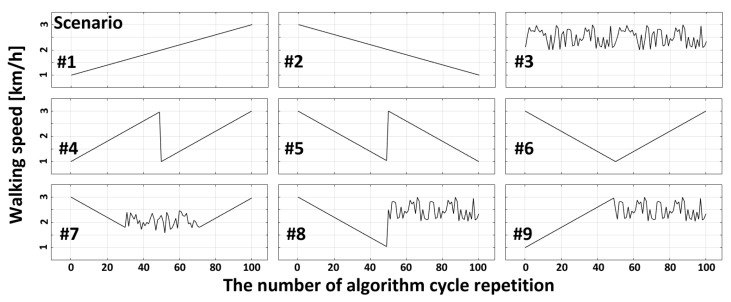
Various exercise scenarios and walking speeds. Each scenario is a combination of scenario elements: linear increase, linear decrease, random with upper and lower boundaries, sudden increase, and sudden decrease. The exercise procedure means exercise from start to end. #1: Linear increase. #2: Linear decrease. #3: Random with upper and lower boundaries. #4: Sudden decrease during linear increase. #5: Sudden increase during linear decrease. #6: Linear increase after linear decrease. #7: Linear decrease—random with upper and lower boundaries—linear increase. #8: Linear decrease—sudden increase—random with upper and lower boundaries. #9: Random with upper and lower boundaries after linear increase.

To evaluate the controllability of the adaptive control algorithm for GDCR, we designed various virtual walking-speed pattern scenarios based on experimental dataset (Walking speed-GDCR and its interpolations). These scenarios consisted of nine combinations of five walking-speed patterns: linear increase, linear decrease, random with upper and lower bounds, sudden increase, and sudden decrease, as shown in Figure 8.

The list below is description for each scenario:

1. The first scenario presents a linear increase in walking speed.
2. The second scenario presents a linear decrease in walking speed.
3. The third scenario presents walking speeds with punctuations within a range.
4. The fourth scenario presents an increasing trend in walking speed followed by a sudden decrease in the middle.
5. The fifth scenario presents a decreasing trend in walking speed followed by a sudden increase in the middle.
6. The sixth scenario presents a V-shaped trend in walking speed.
7. The seventh scenario presents a V-shaped trend in walking speed with a range of random punctuations in the middle.
8. The eighth scenario presents a sudden increase in ranged random punctuations after a linear decrease in walking speed.
9. The ninth scenario presents ranged random punctuations after a linear increase in walking speed.

Also, the list below is description for algorithm processing:

1. For an initial setting, measure the first C_p1_ at 2 km/h of walking speed and 6000 AT.
2. If C_p1_ is recorded as smaller than 0.9, set Gt as 0.01 below C_p1_ (C_p1_—0.01). Otherwise, if C_p1_ was over 0.9, set G_t_ as 0.9.
3. Set new AT for later rehabilitation as AT (G_t_);
4. Do exercise with set AT (G_t_) and measure new GDCR as a result of exercise.
5. Calculate C_pn_ from the measured GDCR.
6. If C_pn_ was smaller than 0.9, set Gt as 0.01 smaller than C_pn_ (C_pn_ − 0.01). Otherwise, if C_pn_ was over 0.9, set Gt as 0.9.
7. Set new AT as AT (G_t_)
8. Repeat process from 4.

The initial setting is from 1 to 3, and the adaptive control algorithm for further rehabilitation is from 4 to 8.

In addition, we prepared control methods to compare the accuracy of the adaptive control method with others;

1. Adaptative, designed method that was previously described;
2. Fixed, which simply fixes the AT at 6000;
3. Simple, which increases or decreases the AT by 100 from the initial value of ‘AT = 6000’ when the GDCR exceeds 0.95 or falls below 0.9, respectively.

These control methods were then applied to the exercise scenarios based on experimental data from subjects. This resulted in a total of 27 simulation results per subject, or 675 in total. The GDCR trends from the various scenarios and algorithms were statistically analyzed. The performance of the control methods was compared using the average error from the target GDCR to 0.9 in all scenarios. The statistical analysis included independent sample Kruskal–Wallis and Mann–Whitney U tests.

**Figure 9:**
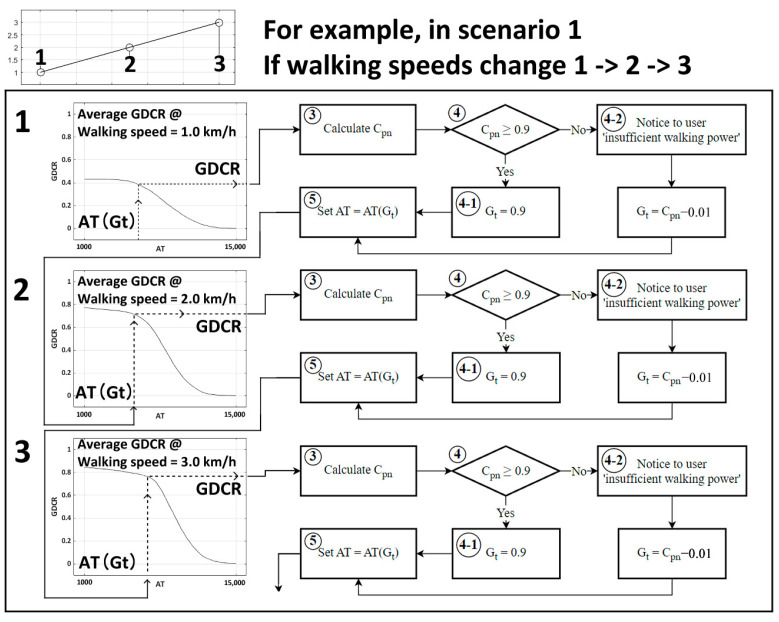
Example of virtual simulation with scenarios. If the walking speed changed from 1.0 km/h, 2.0 km/h, and to 3.0 km/h, the example simulation in scenario one will follow the sequence as shown in this figure.

Simulation with each scenario was ran based on the number of algorithm cycle repetition. For example, if walking speeds change from one to three, as shown in Figure 9, the algorithm measure GDCR is based on the AT(G_target_) at the walking speed = 1.0 km/h. Then, the algorithm return appropriate new AT value ‘AT(G_target_)’ for the next algorithm repetition. The simulation applied new AT(G_target_) to the new GDCR curve at a walking speed = 2.0 km/h and returned a new AT value. These processes repeated until simulation ended. Consequently, the average error to GDCR = 0.9 could be calculated.

### 2.4. Our Goal

Before presenting the results section, we would like to remind readers of what we obtained. Firstly, we calculated the average GDCR for both the left and right legs to determine whether there were any significant differences between the two. Secondly, we computed the average GDCR for different ascending walking speeds to observe the effect of walking speed on GDCR. Thirdly, we examined the impact of GT and AT on GDCR to determine which factor had a dominant effect on controlling GDCR. Finally, we investigated how the adaptive algorithm could sustain a constant GDCR as much as possible under various scenarios.

## 3. Results

### 3.1. Statistical Analysis Results of the Effects of the AT and GT on the GDCR

**Figure 10:**
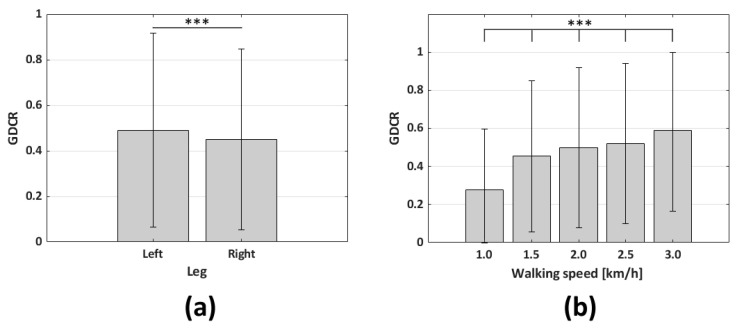
Average GDCR according to legs used and walking speeds. The range of AT was from 1000 to 15,000: (**a**) The relation between the GDCR and used legs (*n* = 246; *** *p* < 0.001); and (**b**) relation between the GDCR and walking speeds (*n* = 246; *** *p* < 0.001).

The statistical analysis in this section was conducted with IBM’s SPSS, and the results are based on how the program works. The average GDCR on the left and right legs exhibited a non-normal distribution (*p* < 0.001, Kolmogorov–Smirnov test), but it differed significantly (*p* < 0.001, *n* = 246, Mann–Whitney U test). The number 246 arises from considering each leg separately, summing 121 from the left and 125 from the right leg, as shown in Table 1. Between the left and right legs, at 0.49 and 0.45, respectively. Although the samples used to examine the correlation between walking speed and GDCR, throughout entire AT range (from 1000 to 15,000), were not normally distributed (*p* < 0.001, Kolmogorov–Smirnov test), the average GDCRs significantly increased (*p* < 0.001, *n* = 246, Kruskal–Wallis test) with walking speeds of 0.27, 0.45, 0.49, 0.52, and 0.59, respectively; 1.0 km/h on the left leg had 47 samples (number of cases × 2 − flaws, 25 × 2 − 3 = 47); 2.0 km/h on the left leg had 49 samples (25 × 2 − 1 = 49); and others had 50 samples (25 × 2 − 0 = 50). Therefore, the sum of samples was 246. These results are shown in Figure 10.

**Figure 11:**
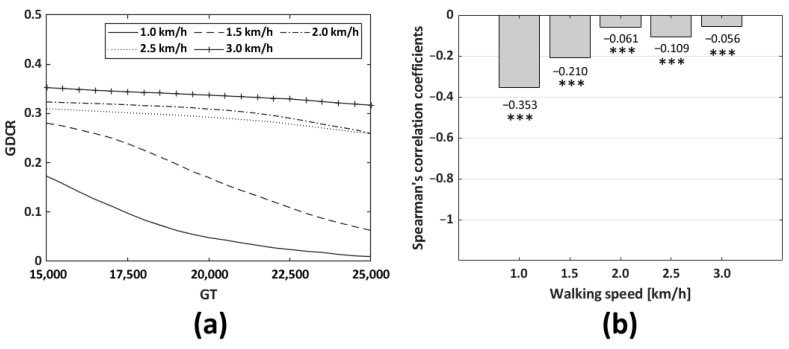
Average GDCR according to the GT and walking speeds. Entire GT range (from 15,000 to 25,000): (**a**) shape of the GDCR curve according to the GT; and (**b**) relation between Spearman’s correlation coefficients and walking speeds (*n* = 441; *** *p* < 0.001 vs. GT, for each walking speed. In sum, *n* = 2205).

**Figure 12:**
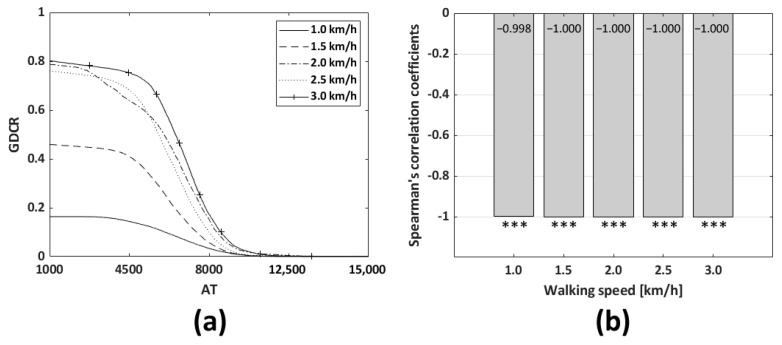
Average GDCR according to the AT and walking speeds: (**a**) shape of the GDCR curve according to the AT. AT more than 15,000 was trimmed in the figure since there were no significant changes in values; and (**b**) relation between Spearman’s correlation coefficients and walking speeds. (*n* = 39,921; *** *p* < 0.001 vs. AT, for each walking speed. In sum, *n* = 199,605).

The dependent variable was GDCR for both, and independent variable were the GT and AT to calculate Spearman correlation coefficients. As a result, the GT was not a significant factor in controlling the GDCR. The Spearman correlation coefficients were −0.353, −0.210, −0.610, −0.109, and −0.056, respectively, and the walking speed were in ascending order of. GT (15,000~25,000) and GDCR according to GT (depending on walking speed) were compared. (*p* < 0.001, *n* = 441 for each walking speed as shown in Table 1, *n* = 2205 in sum). On the other hand, the AT was found to be a significant factor in controlling and reducing the GDCR. The Spearman correlation coefficients were −0.998 at 1 km/h and −1.000 for all other cases. AT (1000~15,000) and GDCR according to AT (depending on walking speed) were compared (*p* < 0.001 *n* = 39,921 for each walking speed as shown in Table 1, *n* = 199,605 in sum). The AT was the most significant factor in controlling the GDCR, and the adaptive control algorithm was designed based on the AT. Figure 11 and Figure 12 show the GDCR according to the GT and AT, respectively, and the corresponding Spearman correlation coefficients.

### 3.2. Algorithm Evaluation

**Figure 13:**
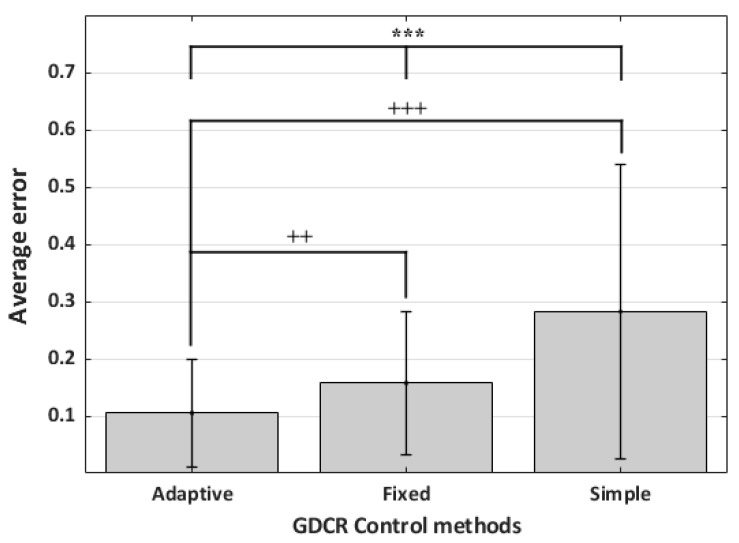
The average error of each control method. (*n* = 225; *** *p* < 0.001, +++ *p* < 0.001, vs. adaptive, ++ *p* < 0.01, vs. adaptive). Errors from the GDCR control algorithm came from a narrower AT range than other results, e.g., those of Figure 10.

In this section, the errors will not be computed from the entire range of AT but from a narrower range, dependent on the control algorithm’s actual performance. The statistical analysis showed that the adaptive control method was the best among three control algorithms (*n* = 675, *p* < 0.001, Kruskal–Wallis test). Error of the result means an absolute value of ‘actual GDCR—0.9.’ The adaptive control method differed significantly from the others (*n* = 675, *p* < 0.001, Mann–Whitney U test). The number of samples, *n*, from one control method and one scenario was 25. Therefore, each control method had 225 (the number of scenarios * the number of participants, 9 × 25 = 225) samples and 675 in sum. The average errors for the adaptive, fixed, and simple control methods were 0.10, 0.16, and 0.28, respectively. Figure 13 shows the results of this analysis.

**Figure 14:**
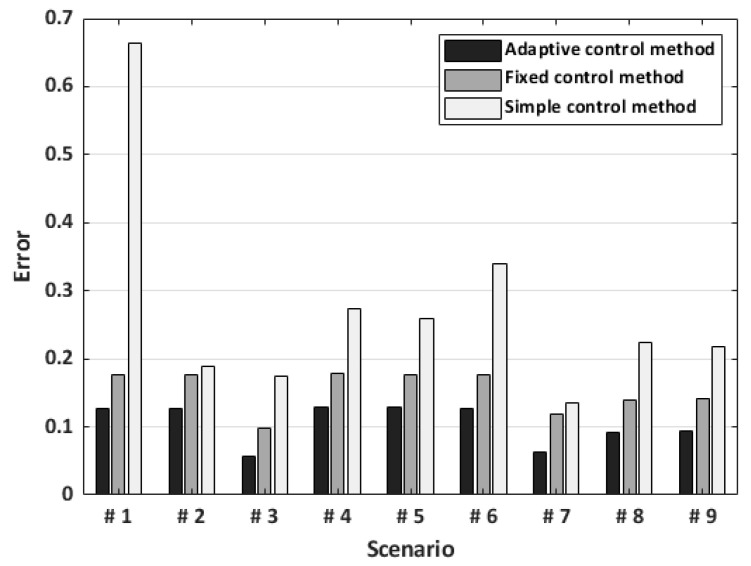
The average error of each control method on every scenario. The N of cases was 25 per each control method in one scenario. The N of cases in one scenario was 75 in total. Errors from GDCR control algorithm came from a narrower AT range, not the full range.

Additionally, the average errors from the experimental dataset were observed for each control method and scenario. The adaptive control method had the best performance in scenario 3, a random scenario, with an average error of 0.06. The highest error was observed in scenario 4, with an average error of 0.14. The fixed control method also had the best results in scenario 3, with the lowest average error of 0.10. The worst performance of the fixed control method was observed in scenario 4, with an average error of 0.18. The simple control method had the lowest average error in scenario 7 (0.13) and the highest average error in scenario 1 (0.66). Figure 14 shows the average errors for all the control methods.

The goal of the adaptive algorithm is to maintain the GDCR close to 0.9; however, why were the average GDCRs for left and right legs 0.49 and 0.45, respectively, in Figure 10? These values came from the full range of AT (from 1000 to 15,000). Therefore, as shown in Figure 12a, the GDCR curve was close to 1.0 at a lower AT and close to 0 at a higher AT. On the contrary, the errors came from algorithm usage. Therefore, errors were calculated from the algorithm control results; consequently, GDCR was usually higher than 0.5 since the control algorithm was designed to sustain GDCR close to 0.9. In short, the GDCRs equal to 0.49 and 0.45 from each leg were from the entire AT range (from 1000 to 15,000), while errors from the GDCR control algorithm came from a narrower AT range.

## 4. Discussion and Conclusions

This study investigated the correlations between the GDCR and various elements used in GDC devices and developed an adaptive GDCR control algorithm to control the GDCR and feedback frequency during exercise using a set AT. The adaptive control algorithm improves the user experience by reducing mental fatigue associated with a higher feedback generation frequency and addresses user uncertainty about the feedback and the functionality of the device when the GDCR is high. However, some readers may still have some confusion from our research. To add more description for better understanding and to avoid confusion, we ask: why the ‘Steady walking speed scenario was not included into virtual scenarios?’ We did not need to include a scenario with constant walking speed. The main purpose of the designed algorithm is to observe how the algorithm can compensate fluctuating GDCR from different walking conditions. At the constant walking speed scenario (1.5 km/h), we expected the AT and GDCR to be invariant with a constant walking speed, and this is actually right. Moreover, we assumed that daily walking speeds would be subtly different in a real-world rehabilitation scenario, so the constant walking speed scenario was altered by including a randomized scenario, i.e., scenario 3.

Previous studies have collected and classified gait factors for diagnosing and rehabilitating patients with Parkinson’s disease, stroke, and other conditions [58,59,60,61,62,63]. Further experiments are needed to determine the effectiveness of the device developed in this study in improving gait symptoms. Other researchers have used IMU systems to quantitatively assess gait [64,65,66,67]. AI has also been applied to assess, evaluate, and extract gait features using IMU systems [43,44,45,46,47]. To the best of our knowledge, there has not been any similar research using an extremely simplified gait detection and assessment system with an associated control algorithm. The device developed in this study uses only one element, AT, to control the GDCR, and does not employ any machine learning methods or AI systems for gait analysis. Therefore, the GDC device and its adaptive control algorithm can roughly but easily detect changes in the user’s condition.

The developed algorithm has good scalability because it uses acceleration around the ankle joint, which can be measured by various devices. For example, the gait rehabilitation algorithm can be used with inertial motion capture systems commonly used in virtual reality production, allowing patients to rehabilitate in a virtual reality environment using a treadmill. The algorithm also has a simple design, making it reproducible and repairable. The device is lightweight, small, and wearable, and can be attached to an ankle brace or another support for ease of use. However, the device already adopted some components to generate a feedback signal needed to be advances. Therefore, additional advanced components such as better and smaller speakers for auditory feedback or lasers for visual feedback can be added to enhance the user experience and portability.

The results of this study confirmed the validity of the existing GDC algorithm after the statistical analysis of the research data. The device and algorithm in this study were designed based on this analysis. Further research is needed to confirm the medical effectiveness of the device and algorithm and to determine their potential for use in clinical settings. In conclusion, the GDC device and adaptive control algorithm developed in this study can provide a rough but easy detection of changes in the user’s condition and have the potential to improve gait symptoms and enhance the user experience.

## Figures and Tables

**Table 1:** Statistical analyses were used to determine the impact of elements used in the GDC algorithm.

Relation	Variable	Number of Subjects	Number of Samples	Statistical Analysis Method
GDCR-Leg	Left leg	25	121	Independent sample Mann–Whitney U test
	Right leg	25	125	
GDCR-Walking speed	1.0 km/h	25	47	Independent sample Kruskal–Wallis test
	1.5 km/h	25	50	
	2.0 km/h	25	49	
	2.5 km/h	25	50	
	3.0 km/h	25	50	
GDCR-GT	1.0 km/h	25	39,921	Spearman’s correlation analysis
	1.5 km/h	25	39,921	
	2.0 km/h	25	39,921	
	2.5 km/h	25	39,921	
	3.0 km/h	25	39,921	
GDCR-AT	1.0 km/h	25	441	
	1.5 km/h	25	441	
	2.0 km/h	25	441	
	2.5 km/h	25	441	
	3.0 km/h	25	441	

## Abbreviations

Parkinson’s Disease (PD); Multi-point IMU (MIMU); Single-point IMU (SIMU); Artificial Intelligence (AI); Machine Learning (ML); Gait Detection and Classification (GDC); Gait Detection and Classification Rate (GDCR); Gyroscopic Threshold (GT); Acceleration Threshold (AT).

## References

[1] Pereira A.P.S., Marinho V., Gupta D., Magalhães F., Ayres C., Teixeira S. (2019). Music Therapy and Dance as Gait Rehabilitation in Patients with Parkinson Disease: A Review of Evidence. J. Geriatr. Psychiatry Neurol..

[2] Lim I., van Wegen E., de Goede C., Deutekom M., Nieuwboer A., Willems A., Jones D., Rochester L., Kwakkel G. (2005). Effects of External Rhythmical Cueing on Gait in Patients with Parkinson’s Disease: A Systematic Review. Clin. Rehabil..

[3] Kearney E., Shellikeri S., Martino R., Yunusova Y. (2019). Augmented Visual Feedback-Aided Interventions for Motor Rehabilitation in Parkinson’s Disease: A Systematic Review. Disabil. Rehabil..

[4] Monteiro-Junior R.S., Cevada T., Oliveira B.R.R., Lattari E., Portugal E.M.M., Carvalho A., Deslandes A.C. (2015). We Need to Move More: Neurobiological Hypotheses of Physical Exercise as a Treatment for Parkinson’s Disease. Med. Hypotheses.

[5] van Duijnhoven H.J.R., Heeren A., Peters M.A.M., Veerbeek J.M., Kwakkel G., Geurts A.C.H., Weerdesteyn V. (2016). Effects of Exercise Therapy on Balance Capacity in Chronic Stroke. Stroke.

[6] Tomlinson C.L., Patel S., Meek C., Herd C.P., Clarke C.E., Stowe R., Shah L., Sackley C.M., Deane K.H., Wheatley K. (2013). Physiotherapy versus Placebo or No Intervention in Parkinson’s Disease. Cochrane Database Syst. Rev..

[7] Gunn H., Markevics S., Haas B., Marsden J., Freeman J. (2015). Systematic Review: The Effectiveness of Interventions to Reduce Falls and Improve Balance in Adults with Multiple Sclerosis. Arch. Phys. Med. Rehabil..

[8] Galea J.M., Mallia E., Rothwell J., Diedrichsen J. (2015). The Dissociable Effects of Punishment and Reward on Motor Learning. Nat Neurosci.

[9] Trotter A.B., Inman D.A. (1968). The Use of Positive Reinforcement in Physical Therapy. Phys. Ther..

[10] Takeda K., Mani H., Hasegawa N., Sato Y., Tanaka S., Maejima H., Asaka T. (2017). Adaptation Effects in Static Postural Control by Providing Simultaneous Visual Feedback of Center of Pressure and Center of Gravity. J. Physiol. Anthropol..

[11] Mirelman A., Herman T., Nicolai S., Zijlstra A., Zijlstra W., Becker C., Chiari L., Hausdorff J.M. (2011). Audio-Biofeedback Training for Posture and Balance in Patients with Parkinson’s Disease. J. NeuroEng. Rehabil..

[12] Hasegawa N., Takeda K., Mancini M., King L.A., Horak F.B., Asaka T. (2020). Differential Effects of Visual versus Auditory Bio-feedback Training for Voluntary Postural Sway. PLoS ONE.

[13] Ginis P., Nieuwboer A., Dorfman M., Ferrari A., Gazit E., Canning C.G., Rocchi L., Chiari L., Hausdorff J.M., Mirelman A. (2016). Feasibility and Effects of Home-Based Smartphone-Delivered Automated Feedback Training for Gait in People with Parkinson’s Disease: A Pilot Randomized Controlled Trial. Park. Relat. Disord..

[14] Dozza M., Chiari L., Chan B., Rocchi L., Horak F.B., Cappello A. (2005). Influence of a Portable Audio-Biofeedback Device on Structural Properties of Postural Sway. J. NeuroEng. Rehabil..

[15] Sienko K.H., Seidler R.D., Carender W.J., Goodworth A.D., Whitney S.L., Peterka R.J. (2018). Potential Mechanisms of Sensory Augmentation Systems on Human Balance Control. Front. Neurol..

[16] Mirelman A., Maidan I., Herman T., Deutsch J.E., Giladi N., Hausdorff J.M. (2011). Virtual Reality for Gait Training: Can It Induce Motor Learning to Enhance Complex Walking and Reduce Fall Risk in Patients with Parkinson’s Disease?. J. Gerontol. Ser. A.

[17] Carpinella I., Cattaneo D., Bonora G., Bowman T., Martina L., Montesano A., Ferrarin M. (2017). Wearable Sensor-Based Bio-feedback Training for Balance and Gait in Parkinson Disease: A Pilot Randomized Controlled Trial. Arch. Phys. Med. Rehabil..

[18] Dozza M., Chiari L., Peterka R.J., Wall C., Horak F.B. (2011). What Is the Most Effective Type of Audio-Biofeedback for Postural Motor Learning?. Gait Posture.

[19] Shen X., Mak M.K.Y. (2014). Balance and Gait Training with Augmented Feedback Improves Balance Confidence in People with Parkinson’s Disease: A Randomized Controlled Trial. Neurorehabil. Neural Repair.

[20] Van den Heuvel M.R.C., Kwakkel G., Beek P.J., Berendse H.W., Daffertshofer A., van Wegen E.E.H. (2014). Effects of Augmented Visual Feedback during Balance Training in Parkinson’s Disease: A Pilot Randomized Clinical Trial. Park. Relat. Disord..

[21] Liao Y.-Y., Yang Y.-R., Wu Y.-R., Wang R.-Y. (2015). Virtual Reality-Based Wii Fit Training in Improving Muscle Strength, Sensory Integration Ability, and Walking Abilities in Patients with Parkinson’s Disease: A Randomized Control Trial. Int. J. Gerontol..

[22] Mhatre P.V., Vilares I., Stibb S.M., Albert M.V., Pickering L., Marciniak C.M., Kording K., Toledo S. (2013). Wii Fit Balance Board Playing Improves Balance and Gait in Parkinson Disease. PM&R.

[23] Baram Y., Aharon-Peretz J., Badarny S., Susel Z., Schlesinger I. (2016). Closed-Loop Auditory Feedback for the Improvement of Gait in Patients with Parkinson’s Disease. J. Neurol. Sci..

[24] Gooßes M., Saliger J., Folkerts A.-K., Nielsen J., Zierer J., Schmoll P., Niepold A., Colbach L., Leemhuis J., Engels L. (2020). Feasibility of Music-Assisted Treadmill Training in Parkinson’s Disease Patients with and without Deep Brain Stimulation: Insights from an Ongoing Pilot Randomized Controlled Trial. Front. Neurol..

[25] Cappozzo A., Della Croce U., Leardini A., Chiari L. (2005). Human Movement Analysis Using Stereophotogrammetry: Part 1: Theoretical Background. Gait Posture.

[26] Mobbs R.J., Phan K., Maharaj M., Rao P.J. (2016). Physical Activity Measured with Accelerometer and Self-Rated Disability in Lumbar Spine Surgery: A Prospective Study. Glob. Spine J..

[27] Phan K., Mobbs R.J. (2016). Long-Term Objective Physical Activity Measurements Using a Wireless Accelerometer Following Minimally Invasive Transforaminal Interbody Fusion Surgery. Asian Spine J..

[28] Sprager S., Juric M.B. (2015). Inertial Sensor-Based Gait Recognition: A Review. Sensors.

[29] Agostini V., Gastaldi L., Rosso V., Knaflitz M., Tadano S. (2017). A Wearable Magneto-Inertial System for Gait Analysis (H-Gait): Validation on Normal Weight and Overweight/Obese Young Healthy Adults. Sensors.

[30] Donath L., Faude O., Lichtenstein E., Pagenstert G., Nüesch C., Mündermann A. (2016). Mobile Inertial Sensor Based Gait Analysis: Validity and Reliability of Spatiotemporal Gait Characteristics in Healthy Seniors. Gait Posture.

[31] Kluge F., Gaßner H., Hannink J., Pasluosta C., Klucken J., Eskofier B.M. (2017). Towards Mobile Gait Analysis: Concurrent Validity and Test-Retest Reliability of an Inertial Measurement System for the Assessment of Spatio-Temporal Gait Parameters. Sensors.

[32] Maffiuletti N.A., Gorelick M., Kramers-de Quervain I., Bizzini M., Munzinger J.P., Tomasetti S., Stacoff A. (2008). Concurrent Validity and Intrasession Reliability of the IDEEA Accelerometry System for the Quantification of Spatiotemporal Gait Parameters. Gait Posture.

[33] Orlowski K., Eckardt F., Herold F., Aye N., Edelmann-Nusser J., Witte K. (2017). Examination of the Reliability of an Inertial Sensor-Based Gait Analysis System. Biomed. Eng./Biomed. Tech..

[34] Rao P.J., Phan K., Maharaj M.M., Pelletier M.H., Walsh W.R., Mobbs R.J. (2016). Accelerometers for Objective Evaluation of Physical Activity Following Spine Surgery. J. Clin. Neurosci..

[35] Tunca C., Pehlivan N., Ak N., Arnrich B., Salur G., Ersoy C. (2017). Inertial Sensor-Based Robust Gait Analysis in Non-Hospital Settings for Neurological Disorders. Sensors.

[36] Mobbs R.J., Katsinas C.J., Choy W.J., Rooke K., Maharaj M. (2018). Objective Monitoring of Activity and Gait Velocity Using Wearable Accelerometer Following Lumbar Microdiscectomy to Detect Recurrent Disc Herniation. J. Spine Surg..

[37] Demonceau M., Donneau A.-F., Croisier J.-L., Skawiniak E., Boutaayamou M., Maquet D., Garraux G. (2015). Contribution of a Trunk Accelerometer System to the Characterization of Gait in Patients with Mild-to-Moderate Parkinson’s Disease. IEEE J. Biomed. Health Inform..

[38] Van Schooten K.S., Pijnappels M., Rispens S.M., Elders P.J.M., Lips P., Daffertshofer A., Beek P.J., Dieën J.H. (2016). van Daily-Life Gait Quality as Predictor of Falls in Older People: A 1-Year Prospective Cohort Study. PLoS ONE.

[39] Del Din S., Galna B., Godfrey A., Bekkers E.M.J., Pelosin E., Nieuwhof F., Mirelman A., Hausdorff J.M., Rochester L. (2019). Analysis of Free-Living Gait in Older Adults with and Without Parkinson’s Disease and with and without a History of Falls: Identifying Generic and Disease-Specific Characteristics. J. Gerontol. Ser. A.

[40] Espinosa H.G., Thiel D.V., Sorell M., Rowlands D. (2020). Can We Trust Inertial and Heart Rate Sensor Data from an APPLE Watch Device?. Proceedings.

[41] Mobbs R.J., Perring J., Raj S.M., Maharaj M., Yoong N.K.M., Sy L.W., Fonseka R.D., Natarajan P., Choy W.J. (2022). Gait Metrics Analysis Utilizing Single-Point Inertial Measurement Units: A Systematic Review. Mhealth.

[42] Prasanth H., Caban M., Keller U., Courtine G., Ijspeert A., Vallery H., von Zitzewitz J. (2021). Wearable Sensor-Based Real-Time Gait Detection: A Systematic Review. Sensors.

[43] Semwal V.B., Gaud N., Lalwani P., Bijalwan V., Alok A.K. (2022). Pattern Identification of Different Human Joints for Different Human Walking Styles Using Inertial Measurement Unit (IMU) Sensor. Artif. Intell. Rev..

[44] Sarshar M., Polturi S., Schega L. (2021). Gait Phase Estimation by Using LSTM in IMU-Based Gait Analysis—Proof of Concept. Sensors.

[45] Trabassi D., Serrao M., Varrecchia T., Ranavolo A., Coppola G., De Icco R., Tassorelli C., Castiglia S.F. (2022). Machine Learning Approach to Support the Detection of Parkinson’s Disease in IMU-Based Gait Analysis. Sensors.

[46] Mundt M., Koeppe A., David S., Witter T., Bamer F., Potthast W., Markert B. (2020). Estimation of Gait Mechanics Based on Simulated and Measured IMU Data Using an Artificial Neural Network. Front. Bioeng. Biotechnol..

[47] Balakrishnan A., Medikonda J., Namboothiri P.K., Natarajan M. (2022). Role of Wearable Sensors with Machine Learning Approaches in Gait Analysis for Parkinson’s Disease Assessment: A Review. Eng. Sci..

[48] Pappas I.P., Popovic M.R., Keller T., Dietz V., Morari M. (2001). A reliable gait phase detection system. IEEE Trans. Neural Syst. Rehabil. Eng..

[49] Mazhar O., Bari A.Z., Faudzi A.A.M. Real-time gait phase detection using wearable sensors. Proceedings of the 2015 10th Asian Control Conference (ASCC).

[50] Li J., Zhou X., Li C., Li W., Zhang H., Gu H. (2016). A Real-Time Gait Phase Detection Method for Prosthesis Control. Assistive Robotics, Proceedings of the 18th International Conference on CLAWAR 2015, Hangzhou, China, 6–9 September 2015.

[51] Goršič M., Kamnik R., Ambrožič L., Vitiello N., Lefeber D., Pasquini G., Munih M. (2014). Online phase detection using wearable sensors for walking with a robotic prosthesis. Sensors.

[52] Yang J., Chen X., Guo H., Zhang Q. Implementation of omnidirectional lower limbs rehabilitation training robot. Proceedings of the 2007 International Conference on Electrical Machines and Systems (ICEMS).

[53] Kim H., Kim J.-W., Ko J. (2021). Gait Disorder Detection and Classification Method Using Inertia Measurement Unit for Augmented Feedback Training in Wearable Devices. Sensors.

[54] Karl K.A., O’Leary-Kelly A.M., Martocchio J.J. (1993). The Impact of Feedback and Self-Efficacy on Performance in Training. J. Organ. Behav..

[55] Burgers C., Eden A., van Engelenburg M.D., Buningh S. (2015). How Feedback Boosts Motivation and Play in a Brain-Training Game. Comput. Hum. Behav..

[56] Wilson K.M., Helton W.S., de Joux N.R., Head J.R., Weakley J.J.S. (2017). Real-Time Quantitative Performance Feedback during Strength Exercise Improves Motivation, Competitiveness, Mood, and Performance. Proc. Hum. Factors Ergon. Soc. Annu. Meet..

[57] Burke J.W., McNeill M.D.J., Charles D.K., Morrow P.J., Crosbie J.H., McDonough S.M. (2009). Optimising Engagement for Stroke Rehabilitation Using Serious Games. Vis. Comput..

[58] Iosa M., Capodaglio E., Pelà S., Persechino B., Morone G., Antonucci G., Paolucci S., Panigazzi M. (2021). Artificial Neural Network Analyzing Wearable Device Gait Data for Identifying Patients with Stroke Unable to Return to Work. Front. Neurol..

[59] Mohan D.M., Khandoker A.H., Wasti S.A., Ismail Ibrahim Ismail Alali S., Jelinek H.F., Khalaf K. (2021). Assessment Methods of Post-Stroke Gait: A Scoping Review of Technology-Driven Approaches to Gait Characterization and Analysis. Front. Neurol..

[60] Nadeau S., Betschart M., Bethoux F. (2013). Gait Analysis for Poststroke Rehabilitation: The Relevance of Biomechanical Analysis and the Impact of Gait Speed. Phys. Med. Rehabil. Clin..

[61] Scheffer C., Cloete T. (2012). Inertial Motion Capture in Conjunction with an Artificial Neural Network Can Differentiate the Gait Patterns of Hemiparetic Stroke Patients Compared with Able-Bodied Counterparts. Comput. Methods Biomech. Biomed. Eng..

[62] Lau H., Tong K., Zhu H. (2009). Support Vector Machine for Classification of Walking Conditions of Persons after Stroke with Dropped Foot. Hum. Mov. Sci..

[63] Zhou Y., Romijnders R., Hansen C., van Campen J., Maetzler W., Hortobágyi T., Lamoth C.J.C. (2020). The Detection of Age Groups by Dynamic Gait Outcomes Using Machine Learning Approaches. Sci. Rep..

[64] Hutabarat Y., Owaki D., Hayashibe M. (2020). Quantitative Gait Assessment with Feature-Rich Diversity Using Two IMU Sensors. IEEE Trans. Med. Robot. Bionics.

[65] Zhou L., Tunca C., Fischer E., Brahms C.M., Ersoy C., Granacher U., Arnrich B. Validation of an IMU Gait Analysis Algorithm for Gait Monitoring in Daily Life Situations. Proceedings of the 2020 42nd Annual International Conference of the IEEE Engineering in Medicine & Biology Society (EMBC).

[66] Gujarathi T., Bhole K. Gait Analysis Using Imu Sensor. Proceedings of the 2019 10th International Conference on Computing, Communication and Networking Technologies (ICCCNT).

[67] Anwary A.R., Yu H., Vassallo M. (2018). An Automatic Gait Feature Extraction Method for Identifying Gait Asymmetry Using Wearable Sensors. Sensors.

